# Ratio maps of T1w/T2w MRI signal intensity do not improve deep-learning segmentation of pediatric brain tumors

**DOI:** 10.1371/journal.pone.0323398

**Published:** 2025-12-22

**Authors:** Daniel Griffiths-King, Timothy Mulvany, Heather Rose, Jan Novak

**Affiliations:** 1 Aston Institute of Health and Neurodevelopment, College of Health and Life Sciences, Aston University, Birmingham, United Kingdom; 2 Medical Physics and Clinical Engineering, Nottingham University Hospitals NHS Trust, Nottingham, United Kingdom; 3 Institute of Cancer and Genomic Sciences, University of Birmingham, Birmingham, United Kingdom; University of Rochester, UNITED STATES OF AMERICA

## Abstract

**Introduction:**

T1w/T2w ratio mapping, combining voxel-wise signal intensities in T1-weighted (T1w) and T2-weighted (T2w) structural MRI, has been used to investigate cortical architecture in the brain, but has also shown promise in tissue discrimination, even in tumor tissue. Given this, we investigate whether the inclusion of these established T1w/T2w ratio maps, or a similar T1w – T2w combined map, can improve performance on a novel task; automated segmentation of tumor tissue in pediatric brain tumor cases from the BraTS-PED 2024 dataset.

**Methods:**

Using the BraTS-PED 2024 dataset (n = 261 pediatric brain tumor patients), we trained and evaluated (with a five-fold cross validation approach) segmentation performance across tumor subregions with nnU-Net, a state-of-the-art deep learning framework. Multiple model configurations were compared; a) a standard baseline model using typical multiparametric MRI (mpMRI, including T1w, T2w, FLAIR and contrast-enhanced T1w MRI) as input modalities and b) an experimental configuration using standard mpMRI inputs plus a T1w/T2w ratio map. Performance was assessed using Dice scores and statistical comparisons with Bonferroni correction to assess he direct ‘added benefit’ of the T1w/T2w ratio maps.

**Results:**

Inclusion of T1w/T2w ratio or the combined maps did not significantly improve segmentation accuracy across any tumor subregion. While minor increases in ET segmentation were observed with the ratio map, these were not statistically significant. Combined maps showed marginal improvements in ET and NET segmentation but reduced performance in CC and ED regions.

**Conclusions:**

Overall, we demonstrate that T1w/T2w ratio maps do not improve deep learning models for segmenting pediatric brain tumor subregions using nnU-Net, despite their strong biophysical basis for tissue discrimination. T hese findings suggest that such data augmentation strategies may not provide added value and highlight the importance of rigorous validation in medical imaging research.

## Introduction

MRI is essential for assessing pediatric brain tumors. Beyond traditional reporting, quantitative MRI analysis identifies in-vivo biomarkers to support diagnosis, predict histopathological status, assess treatment response, and predict prognosis [1]. Accurate tumor boundary delineation is required to quantify biomarkers. Most simply, geometric masks (such as elliptical ROIs of a given size) can be placed within the boundaries of the pathological tissue but there is poor interobserver reliability of these approaches in both pediatric and adult brain tumors [2,3]. Alternatively, tissue deemed to be pathological can be manually delineated. However, individuals with the requisite neuro-radiological expertise to draw these whole-tumor ROIs accurately have limited capacity to conduct this time-consuming process, especially in the context of existing clinical workload. Additionally, even expert-drawn ROIs are subject to inter-/intra-rater variability [4–6]. To avoid manual, labor-intensive methods, automated MRI segmentation via deep learning has gained significant traction.

Segmentation challenges, such as the Brain Tumor Segmentation (BraTS) challenges, promote development of automated approaches which are replicable, generalizable, and accurate, to aid in these tasks. Automated segmentation of pediatric brain tumors has only recently (since 2023) been included as a specific task in the BraTS challenge with contributions by multiple groups providing promising results in terms of segmentation performance [7–9].

Typically, automatic segmentation approaches, including BRaTS, utilizes clinically-acquired MRI including pre/post contrast T1-weighted (T1w & contrast-enhanced T1w (T1w-CE)), T2-weighted (T2w), and T2 Fluid Attenuated Inversion Recovery (T2-FLAIR) as input modalities to deep learning models. These modalities are regularly acquired for standard of care for the clinical management of these patients. Whilst these are usually the only MRI modalities which are clinically available in training data, another way to improve segmentation methods is through novel data augmentation, to generate additional input data for deep learning models (see [10] for further details). This study introduces the T1w/T2w ratio map as a novel model input. Combining T1w and T2w images via a voxel-wise ratio, T1w/T2w mapping provides a high-resolution, non-invasive measure of cortical architecture, distinguishes cortical areas, and minimizes shared field inhomogeneities [11–13].

Historically, T1w/T2w mapping has been used as an in-vivo proxy for myelin content [11] (although recent evidence has called into question the acceptability of this relationship [14,15]) and approximates T1w and T2w relaxometry (R1 & R2 rates) in clinical scenarios where quantitative MRI is impractical [16]. There is limited previous research using these maps but prior research links T1w/T2w ratio with R1/R2 values in tumors [17,18], and can identify non-enhancing regions in glioma [18] – an area where pediatric segmentation methods often struggle [7]. Therefore, the T1w/T2w ratio map may be a valuable novel data augmentation approach for the task of brain tumor segmentation.

Utilizing T1w/T2w ratio maps for automated segmentation may improve tissue discriminability [19], as shown between healthy and glioma tissue for threshold-based segmentation [20]. Similarly, combined T1w-T2w maps, employing voxel-wise scaling rather than strict ratios, outperform individual T1w and T2w MRI in deep-learning segmentation of the claustrum [21]. A combination of existing inputs into deep-learning models, these ratio/combination maps represent a method for data extension to extract additional imaging features.

The current exploratory study evaluates T1w/T2w ratio and combined maps as a novel input to nnU-Net, a leading deep-learning segmentation framework with proven efficacy in brain tumor segmentation [22,23]. nnU-Net automatically adapts preprocessing, network architecture, training, and post-processing, in response to the training data [22]. It is hypothesized that incorporating T1w/T2w maps will improve accuracy of automatic segmentation of pediatric brain tumors using the Brain Tumor Segmentation Pediatrics Challenge (BraTS-PED) 2024 dataset [7].

## Materials and methods

### Data

#### Participants.

The CBTN-CONNECT-DIPGR-ASNR-MICCAI BraTS-PEDs 2024 Challenge dataset is a retrospective cohort consisting of data from n = 464 pediatric patients with high-grade glioma (e.g., high-grade astrocytoma, diffuse midline glioma (DMG) and diffuse intrinsic pontine glioma (DIPG)). It is one of the largest publicly available and well-annotated MRI datasets for this patient group. Only data from the training (n = 261) cohort, where both the MRI and training labels are available, is used in the current study, due to access restrictions on validation/testing cohorts. Further details are published elsewhere [7]. Data are publicly available, and all data were fully anonymized before accessed by the research team as they are shared in a fully anonymized form. Data were obtained through Synapse and MedPerf systems [24] (ID syn511569100029) and Aston University College of Health and Life Sciences Research Ethics Committee (#HLS21041) granted ethical approval for secondary analysis. Data were accessed in May 2024, and authors had no access to identifying data.MRI.

The BraTS-PEDs dataset contains whole-brain, multiparametric MRI (mpMRI) sequences; T1w, T1w-CE, T2w, and T2-FLAIR. MRI data in its publicly shared form was already pre-processed, using the “BraTS Pipeline” (through the Cancer Imaging Phenomics Toolkit (CaPTk) and Federated Tumor Segmentation (FeTS) tool), and anonymized – removing protected DICOM headers and MRI defacing [7]. Briefly, preprocessing involved; conversion of original scan data from DICOM to NIFTI format, co-registration of images to the same anatomical template (the SRI24) and resampling to a 1mm^3^ isotropic resolution.

#### Data annotations – tumor sub-regions.

Reference annotations of four tumor subregions are provided for the training cohort: “enhancing tumor” (ET), “non-enhancing tumor” (NET), “cystic component” (CC) and “edema” (ED). Two additional labels are generated through combinations of subregions, “tumor core” (TC) combining ET, NET, and CC, and “whole tumor” (WT) – the entire tumorous region combining ET, NET, CC and ED. These combinations are created as a simple binary addition of the label masks for the sub-labels. Generating reference annotations involved semi-automated segmentation, iterative refinement/editing of labels, and final review by neuroradiologists [7]).

#### Generating T1w/T2w maps.

*T1w/T2w Ratio Map:* Ratio maps were calculated through normalization/standardization and straightforward division of T1w by T2w images. These were calculated as:


$$T\text{1}w/T\text{2}w\;Ratio\;Map=\frac{T{\text{1}w}_{n}}{T{\text{2}w}_{n}+\alpha }$$

where $T\text{1}w_{n}$ & $T\text{2}w_{n}$ are normalized T1w & T2w images. This follows guidance in [25]. For the purposes of generating the T1w/T2w ratio maps, normalized images $T\text{1}w_{n}$ & $T\text{2}w_{n}$ were calculated by extracting brain tissue masks from T1w images (using FSL [26]), and fitting a Gaussian to the intensities within this mask. Voxel intensities were divided by two times the mean of the Gaussian curve, to normalize the peak to 0.5, with a minimum of 0.

*Combined T1w-T2w Map*: The T1w-T2w combined map was calculated (voxel-wise) as:


$$T\text{1}w-T\text{2}w\;\;Combined\;Map\;=\;\frac{T\text{1}w-\beta \cdot (T\text{2}w)}{T\text{1}w+\beta \cdot (T\text{2}w)}$$

where β is a scaling factor for the purposes of normalization. This echoes the approach in [21]. These were termed a ‘combined’ map as they are not strictly a ratio image. For the purposes of generating the T1w – T2w combined maps, normalization was carried out as follows: SynthSR [27] was used to generate synthetic MRI for each modality (T1w & T2w) to conduct lesion inpainting and produce high quality/contrast images for segmentation. SynthSeg [28] was used for robust automatic segmentation of these synthetic images. SynthSR & SynthSeg were implemented in FreeSurfer v7.3.2. Segmentations of the ventricles (left only) were visually inspected and used as a binary mask to extract mean signal intensity from the raw T1w and T2w images, restricted to within the ventricle. The mean ventricle intensity in the T1w image was divided by the same in the T2w image to calculate the subsequent scaling factor (β) to generate the T1w - T2w combined map.

### Model architectures

The current study uses nnU-Net as the deep learning architecture [22] specifically, the residual encoder variant was used as the benchmarking model, as has been recently recommended (see https://github.com/MIC-DKFZ/nnUNet/blob/master/documentation/resenc_presets.md for further details) [29]. This uses residual blocks in the encoder, which show benefit in brain tumor segmentation tasks [29], where the convolution block’s input is added to the output, preserving information from previous layers.

**Fig 1:**
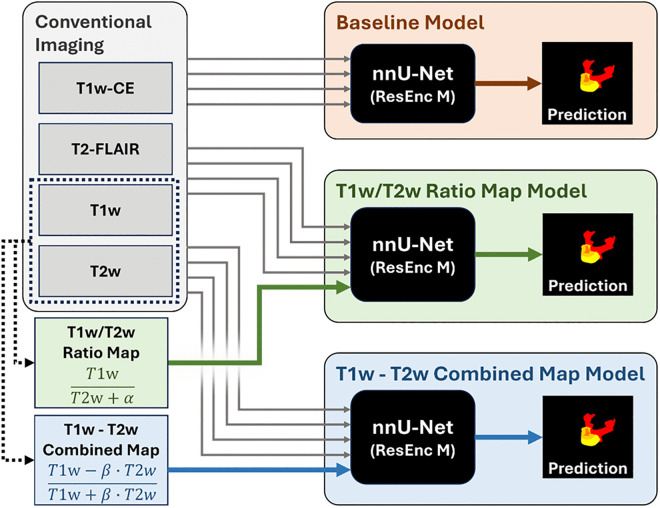
Flow diagram of the current analyses. Workflow of the models tested in the current study, including generation of Ratio and Combined maps.

This study tests three configurations of model inputs; a) a baseline model using the four original mpMRI scans (T1w, T1w-CE, T2-FLAIR and T2w), b) where the T1w/T2w ratio or c) T1w-T2w combined map is included as an additional 5^th^ input channel (see Fig 1).

### Training and evaluation

Training followed nnU-Net’s default methodology, including on-the-fly data augmentation [22], and 5-fold internal-validation with subject-level splits.Each run lasted 100 epochs, selected against the nnU-Net default value based upon initial visual inspection of learning curves. All other training hyperparameters used nnU-Net defaults values. This included a batch size of 2, network optimization using stochastic gradient decent with Nesterov momentum = 0.99, an initial learning rate = 0.01 with a polynomial decay schedule, and a soft Dice and cross-entropy loss function. Models from each fold were ensembled for evaluation per nnU-Net’s default behavior. Reported results reflect performance on the held-out fold during validation.

Performance was evaluated using Dice score (DSC) [7], measuring overlap between automated segmentation and reference labels across the four tumor subregions. When evaluated, a number of tumor labels scored either Dice = 1 or 0. In the case of Dice = 1, this either reflects perfect segmentation or, an empty mask when none of that label exists in the image (i.e., it is correctly identifying that it does not need to segment that tissue label). On visual inspection of our data, where these models scored Dice = 1 it was due to the latter rather than the former. In the case of Dice = 0, this is the case where either no segmentation is attempted (i.e., there can be no overlap between the predicted and ground truth tumor masks if the model does not make a prediction) or that the prediction has no overlapping voxels with the ground truth mask. This is further described in S1 File. In Table 1 we report descriptive data with the cases of Dice = 1 censored, that is to say removed from the data, as this may artificially inflate performance unnecessarily. This is especially important I the current scenario, given that the aim of the current study is to understand if the T1w/T2w ratio maps help improve tissue discrimination, which is not possible in cases that do not have a certain tissue type. The number of cases with either a Dice = 1 or 0 can be found in supporting information (S2 Table).

**Table 1:** Results of segmentation performance for each model during internal validation, across tumor subregion labels.

Model	Dice Score											
	ET			NET			CC			ED		
	Mean	SD	Med.	Mean	SD	Med.	Mean	SD	Med.	Mean	SD	Med.
Baseline	0.550	0.350	0.697	0.783	0.233	0.871	0.271	0.334	0.020	0.174	0.267	0.000
T1w/T2w Ratio Map	0.569	0.344	0.703	0.773	0.250	0.880	0.272	0.325	0.077	0.160	0.254	0.000
Combined T1w-T2w Map	0.553	0.346	0.689	0.785	0.227	0.873	0.266	0. 332	0.005	0.158	0.256	0.000

Note. T1w = T1-weighted MRI, T2w = T2-weighted MRI, ET = Enhancing Tumor, NET = Non-enhancing Tumor, CC = Cystic Component, ED = Edema, Med. = Median, SD = standard deviation, N.B. All reported results are calculated once cases where Dice = 1 have been censored.

Comparative results without censoring can be found in the supplementary materials. In S2 Table we report descriptive data of number of cases with a Dice = 1 or 0 in each of the model outputs. In S3 Table, we report performance for when cases where Dice = 1 are NOT censored. In S4 Table, we report statistical comparisons also without censoring Dice = 1 cases.

To statistically assess segmentation improvement over the baseline model (standard clinical imaging modalities), we compared models additionally incorporating the combined or ratio maps, using a repeated-measures, one-tailed comparisons. As all Dice scores were non-normally distributed, Wilcoxon signed rank tests were adopted.A Bonferroni corrected α_crit_ = 0.0125 addressed multiple comparisons over the 4 tumor subregions. The statistical comparisons censor those cases of Dice = 1, as described above.

### Computational resources

Experiments were conducted with PyTorch (v2.3.0 + CU12.1) on two NVIDIA Quadro RTX6000 GPUs with 24GB VRAM. Ensembling of predictions from differing folds was performed using Python 3.9.19 & NiBabel 5.2.1.

Additional analyzes are reported in supplementary materials.

## Results

**Fig 2:**
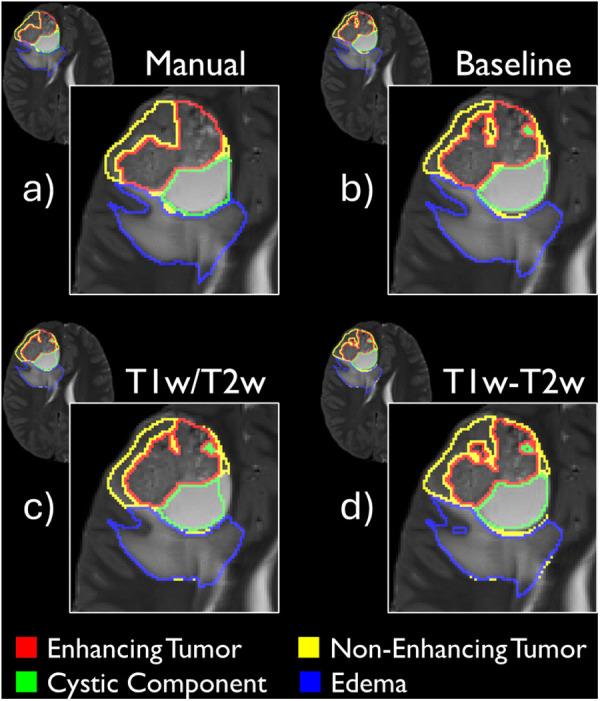
Example segmentation for a single case. Example segmentation executed by a) manual segmentation, b) the baseline model, c) the T1w/T2w ratio map and d) the T1w-T2w combined map. Segmentations are overlaid on the T2w MRI for visualization purposes. Example case was selected as the case with the individual Dice score for the whole tumor (ET + NET + CC + ED) which was closest to the median Dice score, whilst also having each tumor subregion present (e.g., not a case without ED for instance).

**Fig 3:**
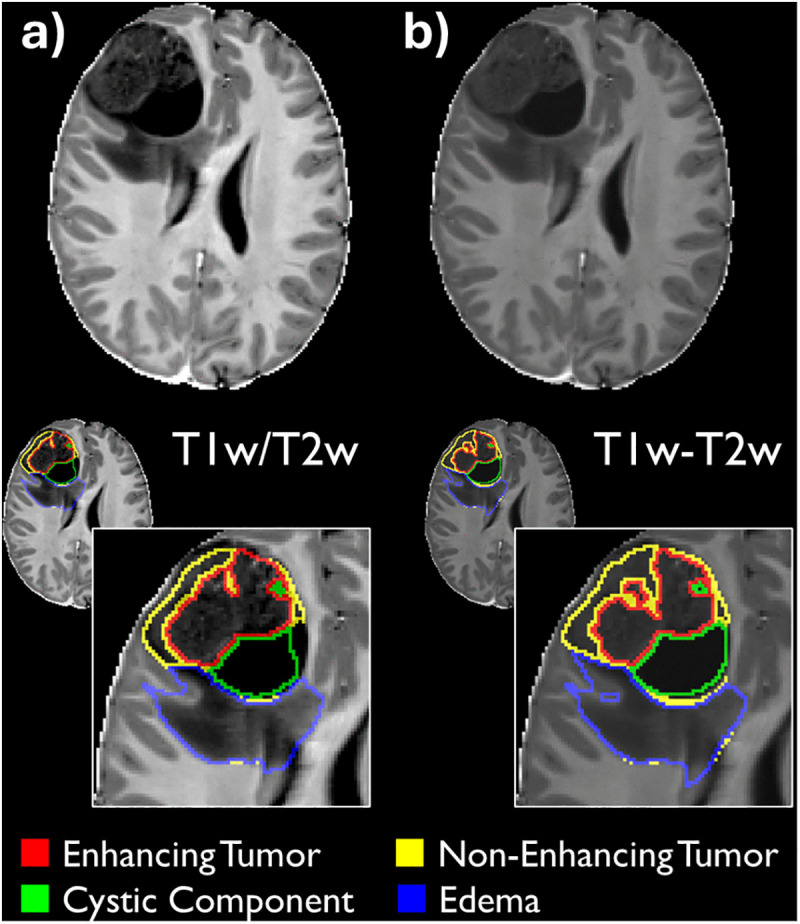
Example of additional input modalities. Examples of a) the T1w/T2w ratio map and **b)** T1w-T2w combined map on the top row, followed by the example segmentations from each model overlaid on their respective ‘additional’ input modality. Example case is the same as selected for Fig 1.

Table 1 presents the segmentation performance accuracy across models and tumor subregions. Table 2 describes the statistical comparisons between the models and baseline. Fig 2 displays a single segmentation comparatively across the three models and the manual segmentation. Fig 3 gives a visualization of both the T1w/T2w ratio map and the T1w-T2w combined map.

**Table 2:** Results of comparisons of performance between models and baseline.

Model	Comparison of Dice Score											
	ET			NET			CC			ED		
	npairs	W	p	npairs	W	p	npairs	W	p	npairs	W	p
T1w/T2w Ratio Map	197	6825	0.407	261	16691	0.589	113	1010	0.986	90	337	0.992
Combined T1w-T2w Map	206	7084	0.509	261	17789	0.215	125	1426	0.499	106	478	0.871

Note. T1w = T1-weighted MRI, T2w = T2-weighted MRI, ET = Enhancing Tumor, NET = Non-enhancing Tumor, CC = Cystic Component, ED = Edema, W = Wilcox (paired) test statistic, N.B. All reported results are calculated once cases where Dice = 1 have been censored, npairs being the valid number of pairs for comparing.

### Baseline model

Performance of the baseline model during internal validation (cross-fold) was highest for the ET & NET labels (${\overset{―}{\text{DSC}}}_{ET}=\text{0}.\text{550},\;{\overset{―}{\text{DSC}}}_{NET}$=0.783). Performance was lowest for the CC and ED subregions (${\overset{―}{\text{DSC}}}_{CC}$=0.271 & ${\overset{―}{\text{DSC}}}_{ED}$=0.174).

### T1w/T2w ratio map model

Performance of the model when T1w/T2w ratio maps were included, was similar to baseline for all tumor subregions during internal validation (${\overset{―}{\text{DSC}}}_{ET}$0.569 vs. 0.550, ${\overset{―}{\text{DSC}}}_{NET}$=0.773 vs. 0.783, ${\overset{―}{\text{DSC}}}_{CC}$=0.272 vs. 0.271, ${\overset{―}{\text{DSC}}}_{ED}$=0.160 vs. 0.174, for ratio map versus baseline models respectively). Accuracy of ET segmentation had the largest increase (+ 0.019) across all tumor subregions and all included models when tested against the baseline model but still did not reach statistical significance. No change in Dice between baseline and the ratio map models was observed to be statistically significant across the tumor subregions (all p > 0.0125).

### Combined T1w-T2w map

When the T1w-T2w combined map was included performance was similar to the baseline model for all tumor subregions during internal validation (${\overset{―}{\text{DSC}}}_{ET}$0.553 vs. 0.550, ${\overset{―}{\text{DSC}}}_{NET}$=0.785 vs. 0.783, ${\overset{―}{\text{DSC}}}_{CC}$=0.266 vs. 0.271, ${\overset{―}{\text{DSC}}}_{ED}$=0.158 vs. 0.174, for ratio map versus baseline models respectively). Whilst ET & NET delineation was marginally improved, larger drops in CC and ED segmentations were observed. No change in Dice between baseline and combine map models was statistically significant (all p > 0.0125).

### Exploratory analysis

In S5 File, to assess specificity and robustness of current segmentation performance to the methods being used to generate the ratio and combined maps, we tested several alternatives, with none providing robust improvements. Results of segmentation performance for each exploratory model are in S6 Table

## Discussion

This study tested whether adding T1/T2 ratio maps improved tumor segmentation in a large, well annotated, pediatric brain tumor MRI dataset. Despite prior literature suggesting potential added benefit, the results showed no statistically significant increase in segmentation performance for ratio or combined approaches.

A small number of previous studies have identified group-level differences in mean T1w/T2w ratio across various pathologies, including adenohypophyseal tumors [30], lung cancer [31] and cerebellar-subtype multiple systems atrophy [32]. Moreover, T1w/T2w ratio as a surrogate for MR relaxometry, had reported benefits in discriminating healthy and tumorous brain tissue [18–20]. This made it a good target for inclusion as an additional input modality for automated tumor segmentation. In this study, inclusion of T1w/T2w ratio maps, alongside standard mpMRI modalities, did not increase segmentation accuracy.

There is a potential assumption that the T1w/T2w ratio maps would only contain redundant information for the purposes of this segmentation task, given that both the T1w and T2w MRI were also used as input modalities across the included models. Whilst the data augmentation technique of generating the T1/T2w ratio maps as novel input channels for the model does not introduce new sematic information, it does in fact change the feature space for learning, so its introduction is not trivial in terms of its downstream effect. Essentially, the approach aimed to generate new representations of the underlying data. Whilst the layers of the network *could* learn to approximate the specific representation of the T1w/T2w Ratio maps, we hypothesized that our approach of ‘hard coding’ this biologically meaningful map improves learning efficiency, embedding this domain knowledge into the input channels rather than placing the burden on the network to ‘learn’ these representations. Therefore, it cannot be assumed from the outset that the new input modality was to be redundant.

However, our data shows that in this case, the additional feature representation offered by this new input modality did not contain complimentary information and instead was redundant. Differences in Dice for specific labels were not statistically significant, and overall, performance on the dataset was low compared to published benchmarks (e.g., BraTS-PEDs 2023 [9]). The interpretation of these findings is that ratio maps do not appear to add any significant additional information, not ascertained through simple additive convolutional differences and the additional computational burden required to generate the maps is not justified. It must be noted though, that the negative findings presented here do not constitute evidence that these maps could not provide complimentary feature representations for future segmentation tasks.

In terms of performance, our models – both the baseline and the model including the T1w/T2w ratio maps performed less favorably than other models in the field. We specifically selected nnU-Net as a strong starting point for challenge-specific optimization and extension [33], with the residual blocks improving performance on brain tumor segmentation tasks (both adults and paediatrics) [29]. nnU-Net featured heavily in the BraTS-PEDs 2023 challenge, of the 9 reported entries, 3 used nnU-Net (or a derivative thereof) to segment pediatric brain tumors, with two instances appearing in the top 3 performing models [9]. These highest performing nnU-Net models adapted the original nnU-Net by self-supervised pretraining integrated with adaptive region-specific loss or an ensemble with Swin UNETR [9]. Adapted nnU-Net models also ranked first in both the adult BraTS 2020 and 2021 challenges [23,33].

In the BraTS-PEDs 2023 challenge, the best performing ET segmentation achieved a Dice of 0.65, higher than this model, but we achieved the same (0.55) performance to the third performing model. Our model specifically also struggled with segmenting both CC and ED, which had lower performance across the tested models. It is unsurprising that the T1/T2 ratio maps did not improve this as previous work only suggested improvements to the broader label of non-enhancing regions in glioma [18]. The reason for broader poor performance in CC and ED is likely due to under-representation of these labels within the training and validation data, not all cases will include these tumor subregions (see our previous BraTS work for further discussion [34]).

It is important to consider alternative reasons for failure to benefit the segmentation process. The T1w/T2w ratio maps may be limited in accuracy, due to technical limitations. For instance, motion could bias the normalization of the T1w and T2w MRI for the calculation of the ratio maps [12], where correction of bias transmit fields assumes the absence of motion. It should also be considered that, in previous research, the T1/T2w ratio maps improved NET tissue discrimination [18], and so there may only be benefit to that tissue type, rather than the multi-label segmentation task we have presented here. It is therefore interesting to note that, whilst non-significant, the NET label did have slight improvement in both mean and median Dice in the T1w/T2w ratio maps.

Previous work which highlighted the benefit of T1w/T2w ratio maps in improved segmentation did not assess the segmentation improvement of including the T1w/T2w ratio maps on top of the existing mpMRI approaches, instead directly comparing to either a T1w only, or T2w only model [21]. Using a single modality baseline models does not allow us to assess whether segmentation improvement is solely due to the inclusion of ratio map inclusion or additional information from the second, T1w or T2w, modality [35]. This is a key strength of the current analysis.

This approach was explored during the development phase of the BraTS-PED 2024 challenge however, it was ultimately abandoned for more effective alternatives. In reporting these null findings, we hope to reduce potential duplication of effort in future challenge contexts and future research efforts by reducing the file-drawer-problem in the medical-imaging field [35].

An important limitation of the current work is the focus upon the BraTS-PED 2024 data. Whilst there is significant benefit in using such a large (relative to the rarity of cases), publicly available dataset, there is only a limited number of tumor types included in the dataset (specifically HGG). Previous work using T1w/T2w ratio maps in brain tumor segmentation has only focused on glioma [18]. It is unclear, and beyond the scope of the current work, to assess how well the current results generalize to other tumor types not represented in the training data. However, the highly limited results shown here may suggest that testing in other tumor types, is likely not a key priority.

Further to this, the BraTS-PED dataset is comprised of contributions from multiple sources/centres including the Children’s Brain Tumor Network, the DMG/DIPG registry, Boston Children’s Hospital, and Yale University [7]. Given the paucity of individual data in terms of which data came from which site, it is unclear whether there may be particular ‘batch-effects’ in the current training data which may require correction via data harmonization.

Overall, combining T1w/T2w imaging modalities does not appear to add value for pediatric brain tumor segmentation when integrated with mpMRI inputs.

## Supporting information

S1 File Dealing with ‘Failed’ cases. (DOCX)

S2 Table Number (n) of cases with a Dice Score of 1 or 0. (DOCX)

S3 Table Results of segmentation performance for each model during internal validation, across subregions without censoring cases where Dice = 1. (DOCX)

S4 Table Results of comparisons of performance between these models and baseline without censoring cases where Dice = 1. (DOCX)

S5 File Exploratory analysis. (DOCX)

S6 Table Results of segmentation performance for each exploratory model during internal validation, across tumor subregion labels. (DOCX)
