# Supplementary material for: Ratio maps of T1w/T2w MRI signal intensity do not improve deep-learning segmentation of pediatric brain tumors
Source: PLoS One. 2025 Dec 22;20(12):e0323398. doi: 10.1371/journal.pone.0323398 (PMC12721524; doi:10.1371/journal.pone.0323398)
Supplement: S1 File — (DOCX) [file pone.0323398.s001.docx]

**Automatic Segmentation of Pediatric Brain Tumors using Ratio Maps of T1w/T2w MRI Signal Intensity**

**S1 Supporting Information. Dealing with ‘Failed’ cases**

When evaluated, a number of tumor labels scored either Dice = 1 or 0. In the case of Dice = 1, this either reflects perfect segmentation or, an empty mask when none of that label exists in the image (i.e. it is correctly identifying that it does not need to segment that tissue label). On visual inspection of our data, where these models scored Dice = 1 it was due to the latter rather than the former. In the case of Dice = 0, this is the case where ether no segmentation is attempted (i.e. there can be no overlap between the predicted and ground truth tumor masks if the model does not make a prediction) or that the prediction has no overlapping voxels with the ground truth mask, In Table 1 we report descriptive data with the cases of Dice = 1 censored, that is to say removed, from the data, as this may artificially inflate performance unnecessarily. This is especially true given that the aim of the current study is to understand if the T1w/T2w ratio maps help improve tissue discrimination, which is not possible in cases that do not have a certain tissue type. The statistical comparisons described below also censor those cases of Dice = 1.

Comparative results without censoring can be found in the supplementary materials. In Table S1 we report descriptive data of number of cases with a Dice=1 or 0 in each of the model outputs. In S2, we report performance for when cases where Dice = 1 are NOT censored. In Table S3, we report statistical comparisons also without censoring Dice = 1 cases.
