# Supplementary material for: Ratio maps of T1w/T2w MRI signal intensity do not improve deep-learning segmentation of pediatric brain tumors
Source: PLoS One. 2025 Dec 22;20(12):e0323398. doi: 10.1371/journal.pone.0323398 (PMC12721524; doi:10.1371/journal.pone.0323398)
Supplement: S2 Table — (DOCX) [file pone.0323398.s002.docx]

**Automatic Segmentation of Pediatric Brain Tumors using Ratio Maps of T1w/T2w MRI Signal Intensity**

**S2 Table. Number (n) of cases with a Dice Score of 1 or 0**

| Model | Label | | | | | | | | | | | | |
| --- | --- | --- | --- | --- | --- | --- | --- | --- | --- | --- | --- | --- | --- |
|  | ET | |  | NET | |  | CC | |  | | ED | |  |
|  | Dice=1 | Dice=0 |  | Dice=1 | Dice=0 |  | Dice=1 | Dice=0 | |  | Dice=1 | Dice=0 |  |
| Baseline | 50 | 46 |  | 0 | 2 |  | 129 | 58 | |  | 145 | 70 |  |
| T1w/T2w Ratio Map | 60 | 42 |  | 0 | 4 |  | 142 | 52 | |  | 159 | 59 |  |
| Combined T1w-T2w Map | 50 | 45 |  | 0 | 4 |  | 125 | 66 | |  | 138 | 76 |  |
| Note. T1w = T1-weighted MRI, T2w = T2-weighted MRI, ET = Enhancing Tumor, NET = Non-enhancing Tumor, CC = Cystic Component, ED = Edema, | | | | | | | | | | | | | |
