# Supplementary material for: Ratio maps of T1w/T2w MRI signal intensity do not improve deep-learning segmentation of pediatric brain tumors
Source: PLoS One. 2025 Dec 22;20(12):e0323398. doi: 10.1371/journal.pone.0323398 (PMC12721524; doi:10.1371/journal.pone.0323398)
Supplement: S3 Table — (DOCX) [file pone.0323398.s003.docx]

**Automatic Segmentation of Pediatric Brain Tumors using Ratio Maps of T1w/T2w MRI Signal Intensity**

**S3 Table. Results of segmentation performance** for each model during internal validation, across subregions **without censoring cases where Dice=1**

| Model | Dice Score | | | | | | | | | | | |
| --- | --- | --- | --- | --- | --- | --- | --- | --- | --- | --- | --- | --- |
|  | ET | | | NET | | | CC | | | ED | | |
|  | Mean | SD | Med. | Mean | SD | Med. | Mean | SD | Med. | Mean | SD | Med. |
| Baseline | 0.636 | 0.361 | 0.781 | 0.783 | 0.233 | 0.871 | 0.632 | 0.436 | 0.908 | 0.633 | 0.448 | 1 |
| T1w/T2w Ratio Map | 0.668 | 0.353 | 0.806 | 0.773 | 0.251 | 0.880 | 0.668 | 0.425 | 1 | 0.672 | 0.440 | 1 |
| Combined T1w-T2w Map | 0.639 | 0.358 | 0.785 | 0.785 | 0.228 | 0.873 | 0.618 | 0.439 | 0.882 | 0.603 | 0.456 | 1 |
| Note. T1w = T1-weighted MRI, T2w = T2-weighted MRI, ET = Enhancing Tumor, NET = Non-enhancing Tumor, CC = Cystic Component, ED = Edema, Med. = Median, SD = standard deviation | | | | | | | | | | | | |
