# Supplementary material for: Ratio maps of T1w/T2w MRI signal intensity do not improve deep-learning segmentation of pediatric brain tumors
Source: PLoS One. 2025 Dec 22;20(12):e0323398. doi: 10.1371/journal.pone.0323398 (PMC12721524; doi:10.1371/journal.pone.0323398)
Supplement: S4 Table — (DOCX) [file pone.0323398.s004.docx]

**Automatic Segmentation of Pediatric Brain Tumors using Ratio Maps of T1w/T2w MRI Signal Intensity**

**S4 Table. Results of comparisons of performance** between these models and baseline **without censoring cases where Dice=1**

| Model | Comparison of Dice Score | | | | | | | | | | | |
| --- | --- | --- | --- | --- | --- | --- | --- | --- | --- | --- | --- | --- |
|  | ET | | | NET | | | CC | | | ED | | |
|  | npairs | W | p | npairs | W | p | npairs | W | p | npairs | W | p |
| T1w/T2w Ratio Map | 261 | 9236 | 0.078 | 261 | 16691 | 0.589 | 261 | 2682 | 0.295 | 261 | 2066 | 0.146 |
| Combined T1w-T2w Map | 261 | 7952 | 0.508 | 261 | 17788 | 0.215 | 261 | 2018 | 0.741 | 261 | 1098 | 0.959 |
| Note. T1w = T1-weighted MRI, T2w = T2-weighted MRI, ET = Enhancing Tumor, NET = Non-enhancing Tumor, CC = Cystic Component, ED = Edema, W = Wilcox (paired) test statistic | | | | | | | | | | | | |
