# Supplementary material for: Ratio maps of T1w/T2w MRI signal intensity do not improve deep-learning segmentation of pediatric brain tumors
Source: PLoS One. 2025 Dec 22;20(12):e0323398. doi: 10.1371/journal.pone.0323398 (PMC12721524; doi:10.1371/journal.pone.0323398)
Supplement: S5 File — (DOCX) [file pone.0323398.s005.docx]

**Automatic Segmentation of Pediatric Brain Tumors using Ratio Maps of T1w/T2w MRI Signal Intensity**

**S5 Supporting Information. Exploratory Analysis**

To assess specificity and robustness of current segmentation performance to the methods being used to generate the ratio and combined maps, we tested a number of similar methods.

To assess whether results were specific to the combination of modalities in the T1w - T2w maps, we additionally generated two additional combination maps using both T2wFLAIR and T1wCE images as follows:

$$T2FLAIR-T2w Combined Map = \frac{T2{FLAIR}_{n}-T2w_{n}}{T2{FLAIR}_{n}+T2w_{n}}$$

$$T1w-T1wCE Combined Map = \frac{T1w_{n}-T1{wCE}_{n}}{T1w_{n}+T1{wCE}_{n}}$$

where for each modality, normalization was conducted using the same method as the T1w/T2w Ratio Map. To assess whether results were robust to the normalization approach used, we recalculated the Combined T1w/T2w Map using the Gaussian normalization approach used in the T1w/T2w Ratio Map (rather than the scaling factor (SF) method) resulting in the following additional map:

$$T1w/T2w (normalised) Ratio Map = \frac{T1w_{n}-T2w_{n}}{T1w_{n}+T2w_{n}}$$

This differs from the previous combined maps primarily in terms of the way in which normalization is applied. These three additional maps were assessed in the same way as those in the main text for direct comparison.

Table S3 displays the performance of these additional maps in comparison to the baseline model when assessed in an internal validation approach (cross-fold validation). No apparent improvements are seen, either through qualitative inspection or statistically except for the T1w -T1wCE Combined Map. Table S4 shows the results of statistical comparisons for these additional maps. It is important to note, that these are using the Dice = 1 censoring applied in the main text and discussed further in above supplementary materials.

A Wilcoxon signed-rank test indicated that inclusion of this map in the segmentation model resulted in a significant increase in segmentation accuracy of the enhancing tumor region (${\bar{\mathrm{DSC}}}_{ET}$= 0.566 vs 0.550, *z* = 10112, *p* = 0.0039).
