# Supplementary material for: Ratio maps of T1w/T2w MRI signal intensity do not improve deep-learning segmentation of pediatric brain tumors
Source: PLoS One. 2025 Dec 22;20(12):e0323398. doi: 10.1371/journal.pone.0323398 (PMC12721524; doi:10.1371/journal.pone.0323398)
Supplement: S6 Table — (DOCX) [file pone.0323398.s006.docx]

**Automatic Segmentation of Pediatric Brain Tumors using Ratio Maps of T1w/T2w MRI Signal Intensity**

**S6 Table.** Results of segmentation performance for each exploratory model during internal validation, across tumor subregion labels

| Model | DICE Score | | | | | | | | | | | |
| --- | --- | --- | --- | --- | --- | --- | --- | --- | --- | --- | --- | --- |
|  | ET | | | NET | | | CC | | | ED | | |
|  | Mean | SD | Med. | Mean | SD | Med. | Mean | SD | Med. | Mean | SD | Med. |
| Baseline | 0.550 | 0.350 | 0.697 | 0.783 | 0.233 | 0.871 | 0.271 | 0.334 | 0.020 | 0.174 | 0.267 | 0.000 |
| T2FLAIR - T2w Combined Map | 0.564 | 0.341 | 0.705 | 0.785 | 0.225 | 0.871 | 0.260 | 0.331 | 0.010 | 0.165 | 0.262 | 0.000 |
| T1w - T1wCE Combined Map | 0.566 | 0.340 | 0.703 | 0.787 | 0.225 | 0.875 | 0.268 | 0.333 | 0.025 | 0.165 | 0.258 | 0.000 |
| T1w/T2w (normalized) Ratio Map | 0.555 | 0.346 | 0.689 | 0.783 | 0.229 | 0.878 | 0.251 | 0.330 | 0.001 | 0.181 | 0.273 | 0.000 |
| Note. T1w = T1-weighted MRI, T2w = T2-weighted MRI, ET = Enhancing Tumour, NET = Non-enhancing Tumour, CC = Cystic Component, ED = Edema, Med. = Median | | | | | | | | | | | | |
